# Mitochondrial Dysfunction in Parkinson’s Disease: From Mechanistic Insights to Therapy

**DOI:** 10.3389/fnagi.2022.885500

**Published:** 2022-06-20

**Authors:** Xiao-Yan Gao, Tuo Yang, Ying Gu, Xiao-Hong Sun

**Affiliations:** ^1^Department of Neurology, The Fourth Affiliated Hospital of China Medical University, Shenyang, China; ^2^Science Experiment Center, China Medical University, Shenyang, China

**Keywords:** Parkinson’s disease, mitochondrial dysfunction, bioenergetics, mitochondrial quality control, therapy

## Abstract

Parkinson’s disease (PD) is one of the most common neurodegenerative movement disorders worldwide. There are currently no cures or preventative treatments for PD. Emerging evidence indicates that mitochondrial dysfunction is closely associated with pathogenesis of sporadic and familial PD. Because dopaminergic neurons have high energy demand, cells affected by PD exhibit mitochondrial dysfunction that promotes the disease-defining the loss of dopaminergic neurons in the substantia nigra pars compacta (SNpc). The mitochondrion has a particularly important role as the cellular “powerhouse” of dopaminergic neurons. Therefore, mitochondria have become a promising therapeutic target for PD treatments. This review aims to describe mitochondrial dysfunction in the pathology of PD, outline the genes associated with familial PD and the factors related to sporadic PD, summarize current knowledge on mitochondrial quality control in PD, and give an overview of therapeutic strategies for targeting mitochondria in neuroprotective interventions in PD.

## Introduction

Parkinson’s disease (PD) is one of the most common neurodegenerative movement disorders worldwide. The pathological hallmarks are the loss of dopaminergic neurons in the substantia nigra pars compacta (SNpc) and the formation of Lewy bodies (LBs), of which a major component is the protein α-synuclein (α-syn). Clinical features of PD include motor symptoms such as rigidity, bradykinesia, postural deformities and instability, gait dysfunction, and resting tremor; non-motor symptoms include autonomic dysfunction, sleep disorders, cognitive abnormalities, and constipation, see the reviews by Balestrino and Schapira (2020) and Bloem et al. (2021). The current treatments include pharmacologic therapies and surgical interventions such as deep brain stimulation; however, there are currently no cures or preventative treatments for PD.

Most PD cases are sporadic and approximately 10% of cases are inherited (Shadrina and Slominsky, 2021). With the recent discovery of several causative monogenetic mutations, academic interest in PD has grown significantly. Evidence indicates that mitochondria play a crucial role in neurodegenerative diseases. The selective degeneration of dopaminergic neurons in the SNpc leads to striatal dopamine (DA) depletion, causing the classic motor symptoms of PD (Yoo et al., 2018). Because dopaminergic neurons have high energy demand, the mitochondrion has a particularly important role as the cellular “powerhouse.” Therefore, although the etiology of PD is complex, mitochondrial dysfunction remains an important step in the pathogenesis of the disease. Furthermore, mitochondrial dysfunction is a feature common to the monogenic and sporadic forms of PD.

Mitochondria are vital and highly dynamic organelles that provide energy to cells. Mitochondrial dysfunction can lead to bioenergetic deficiency, generation of reactive oxygen species (ROS), alteration of mitochondrial dynamics, and induction of oxidative stress-induced apoptosis. In this review, we focus on mitochondrial dysfunction in the pathology of PD, outline the genes associated with familial PD and the factors related to sporadic PD, summarize current knowledge on the role of mitochondrial quality control of the disorder, and give an overview of therapeutic strategies for targeting mitochondria in neuroprotective interventions for PD.

## Genes Associated with Mitochondrial Dysfunction in Parkinson’s Disease

The first PD-associated gene, synuclein alpha (*SNCA*), was discovered in 1997 (Polymeropoulos et al., 1997). With the development of gene technologies and population studies, more than 20 genes have been discovered in monogenic forms of PD; these genes include *SNCA*, *LRRK2*, *PARKIN*, *PINK1*, and *DJ-1*. Some of these genes are associated with mitochondrial dysfunction (Table 1).

**TABLE 1:** Mitochondrial dysfunction-related genes in Parkinson’s disease.

Mode	Gene	Protein name	PARK locus	Gene locus	Age at onset
Autosomal dominant	*SNCA* (Polymeropoulos et al., 1996)	Alpha-synuclein	PARK 1/4	4q21-23	Early or late
	*LRRK2* (Funayama et al., 2002)	Leucine-rich repeat kinase 2	PARK 8	12p11.2-q13.1	Early or late
	*VPS35* (Paravicini et al., 1992)	Vacuolar protein sorting 35	PARK 17	16q11.2	Late
	*CHCHD2* (Funayama et al., 2015)	Coiled-helix-coiled-helix domain containing 2	PARK 22	7q11.2	Early
Autosomal recessive	*PRNK* (Matsumine et al., 1997)	Parkin	PARK 2	6q25.2-27	Juvenile (>20 years) Early (20–40 years)
	*PINK1* (Valente et al., 2001)	PTEN-induced putative kinase 1	PARK 6	1p35-p36	Juvenile (>20 years) Early (20–40 years)
	*DJ-1* (van Duijn et al., 2001)	DJ-1 (parkinsonism associated deglycase)	PARK 7	1p36	Early
	*ATP13A2* (Ramirez et al., 2006)	ATPase 13A2	PARK 9	1p36	Early
	*PLA2G6* (Kinghorn et al., 2015)	Calcium-independent phospholipase A2β (iPLA2β)	PARK 14	22q13.1	Early (Juvenile dystonia-parkinsonism)
	*FBXO7* (Burchell et al., 2013)	F-box domain of protein 7	PARK 15	22q12-13	Early

### Mitochondrial Dysfunction in Autosomal Dominant Parkinson’s Disease

#### SNCA

α-Synuclein is a presynaptic protein composed of 140 amino acid polypeptides and encoded by *SNCA*. It is implicated in several neurodegenerative diseases collectively known as synucleinopathies, including PD (Spillantini et al., 1997). *SNCA* missense mutations (A30P, E46K H50Q, G51D, A53E, and A53T) and whole locus duplication or triplication are related to autosomal dominant PD (Ibanez et al., 2004). Early reports have shown that the presence of the promoter polymorphic variant of *SNCA* increases the risk of sporadic PD.

Wild type α-syn overexpression or mutation causes mitochondrial fragmentation, mitochondrial membrane potential reduction, mitochondrial axonal transport inhibition, reactive oxygen species (ROS) production, and Ca^2+^ homeostasis imbalance at mitochondria-associated membranes (MAMs), see Melo et al.’s (2018) review. Furthermore, α-syn has a mitochondrial targeting sequence (MTS) at the N-terminal and is localized to the outer mitochondrial membrane (OMM), intermembrane space (IMS), inner mitochondrial membrane (IMM) (Vicario et al., 2019). α-Syn regulates mitochondrial function through maintenance mitochondrial morphology and regulating complex I activity (Vicario et al., 2019). α-Syn can even be imported into mitochondria under specific conditions. α-Syn is localized to a subcellular structure between the endoplasmic reticulum and mitochondria called MAMs (Guardia-Laguarta et al., 2014). A pathogenic α-syn mutation was shown to increase the number of MAMs and enhance mitochondrial Ca^2+^ transients, suggesting that impairment of α-syn and Ca^2+^ cross-talk in MAMs is involved in the pathogenesis of PD (Cali et al., 2019). The pathogenicity of α-syn is associated with its aggregation. Accumulation of α-syn is directly related to increased Ca^2+^ levels in mitochondria (Erustes et al., 2021), which increases oxidative stress and cytochrome C release. Reduced levels of voltage dependent anion channel protein 1 (VDAC1) in the SNpc neurons of sporadic PD patients was associated with aggregations of α-syn (Chu et al., 2014), which may have been mediated by the activation of the mitochondrial permeability transition pore (mPTP). Aggregated α-syn affects various cellular functions, including mitochondrial function, autophagy, protein transcription, and synaptic function (Tang et al., 2021; Zhang et al., 2022). Transcriptomic analysis of induced pluripotent stem cells (iPSCs) carrying an A53T *SNCA* mutation and a triplication discovered that the expression of mitochondrial function-related genes was perturbed (Zambon et al., 2019), which could explain the observed reduction in mitochondrial respiration, aberrant mitochondrial morphology, and decreased mitochondrial membrane potential. Interestingly, α-syn can also impair mitochondrial function by reducing the expression of peroxisome proliferator-activated receptor gamma coactivator 1-α (PGC1α) and related target genes (Wilkaniec et al., 2021). For more detail on α-syn and mitochondrial dysfunction in PD, please see the excellent review by Rocha et al. (2018).

#### LRRK2

Leucine-rich repeat kinase 2 (LRRK2) is a multifunctional protein complex (268 kDa) composed of 2527 amino acids that participates in a wide range of cellular activities. There are two main structures related to the enzymatic core: a serine-threonine kinase domain and a GTPase domain. Although the function of LRRK2 has not been completely elucidated, studies have shown that the protein is involved in regulation of mitochondrial dynamics and mitochondrial quality control (Taymans et al., 2015). Mutations in *LRRK2* cause the late-onset autosomal dominant form of PD, which represents the most common familiar PD, see Taylor and Alessi’s (2020) review. PD patients with *LRRK2* mutations show clinical traits and pathological features indistinguishable from those of sporadic PD cases (Tolosa et al., 2020). Nine *LRRK2* missense mutations have confirmed pathogenicity, including G2019S, Y1699C, R1441C/G/H, N1437H, I2020T, G2385R, and R1628P, see Lunati et al.’s (2018) review.

Increasing evidence has confirmed the key role of LRRK2 in mitochondrial function. LRRK2 is located mainly in the cytoplasm, although approximately 10% of LRRK2 is located to the mitochondria and regulates mitochondrial function (Biskup et al., 2006). LRRK2 partially colocalizes with regulators of mitochondrial fission/fusion and may enhance dynamin-related protein 1 (DRP1) mitochondrial translocation through interaction with DRP1, indicating that it plays an indirectly role of regulating mitochondrial dynamics (Stafa et al., 2014). More mitochondrial fragmentation was also observed in the mouse embryonic fibroblast cells (MEFs) expressing G2019S mutant LRRK2 (Toyofuku et al., 2020). In contrast, another study reported enhanced mitochondrial elongation and interconnectivity in fibroblasts harboring the G2019S mutation (Mortiboys et al., 2010). *LRRK2* mutations in DA neurons were associated with an increased susceptibility to oxidative stress (Cooper et al., 2012). Furthermore, a decreased basal oxygen consumption rate and increased mtDNA damage were observed in G2019S mutation models (Gonzalez-Hunt et al., 2020). Mice overexpressing WT or G2019S-mutant LRRK2 exhibited impaired microtubule assembly and decreased levels of free tubulin in brain tissue extracts, indicating inhibited mitochondrial transport (Lin et al., 2009; Parisiadou et al., 2009). The G2019S mutation interferes with its kinase activity, causing mtDNA dyshomeostasis that results in impaired mitophagy (Delcambre et al., 2020).

#### VPS35

Vacuolar protein sorting 35 (VPS35), composed of 796 amino acids, is a core component of the retromer complex and participates in endosomal-lysosomal transport. *VPS35* mutations cause late-onset autosomal dominant PD. The missense mutation D620N has been identified in white families with autosomal dominant PD; in contrast, *VPS35* mutations are rare in Asian populations, except for Japanese populations (Ando et al., 2012). While the discovery of disease-related mutations in *VPS35* indicates that retromer complex dysfunction is a component of PD, our understanding of the molecular and cellular mechanisms of neurodegeneration is still limited. Expression of D620N VPS35 in cortical neurons led to impaired neurite outgrowth and induced dopaminergic neurodegeneration in a novel virus-mediated rat model (Tsika et al., 2014). Neuronal vulnerability and sensitivity to 1-methyl-4-phenylpyridinium (MPP^+^) and rotenone was significantly increased in the overexpression of WT and D620N VPS35 models (Bi et al., 2013; Wang et al., 2014). A landmark study showed that VPS35 mediated vesicle transport *via* formation of mitochondrial-derived vesicles (MDVs) and played an important role in mediating transport of the vesicles from mitochondria to peroxisomes and lysosomes (Sugiura et al., 2014; Picca et al., 2020). PD-related *VPS35* mutations resulted in mitochondrial fragmentation and neuronal loss in neurons *in vivo* and *in vitro* (Wang et al., 2016). Mitochondrial dysfunction and cell death caused by VPS35 can be prevented by inhibition of mitochondrial fission protein dynamin-like protein 1 (DLP1) (Wang et al., 2016); it was reported that VPS35 was recruited to mitochondrial vesicles, silencing VPS35 and causing a significant reduction in the delivery of mitochondrial-anchored protein ligase (MAPL) to peroxisomes (Braschi et al., 2010). In addition, VPS35 is a putative modulator of mitochondrial dynamics; VPS35 regulates mitofusin2 (MFN2) by controlling the transport of mitochondrial E3 ubiquitin ligase 1 (MUL1) to the OMM, which affects mitochondrial fusion (Wu et al., 2020). For more information about VPS35 and mitochondria in PD pathophysiology, please refer to Cutillo et al.’s (2020) review.

#### CHCHD2

Coiled-helix-coiled-helix domain containing 2 (CHCHD2) is a mitochondrial protein located in the IMS (Cornelissen et al., 2020). CHCHD2 is necessary for oxidative phosphorylation (OXPHOS) and promotes mitochondrial oxygen consumption (Baughman et al., 2009). Missense mutations are associated with early-onset autosomal dominant PD with a typical phenotype. A large cohort study identified nine rare exonic variants of CHCHD2 in PD cases; eight of the nine exon variants were found in the MTS of CHCHD2, indicating that rare variants of CHCHD2 may link with mitochondrial dysfunction of PD pathogenesis (Ogaki et al., 2015).

Although the function of CHCHD2 has not been fully elucidated, studies have reported that CHCHD2 expression affected the activity of complex I and complex IV, mitochondrial biogenesis, mitochondrial stability, and cristae structure (Baughman et al., 2009; Wei et al., 2015; Meng et al., 2017). Silencing *CHCHD2* reduced the activity of complex I and complex IV, indicating that CHCHD2 may promote mitochondrial respiration (Baughman et al., 2009). A recent study showed that CHCHD2 regulated mitochondrial morphology by fine regulating the levels of optic atrophy 1 (OPA1) (Liu et al., 2020). Some mitochondria showed a reduced number of cristae and altered cristae structure in the muscles of adult Drosophila harboring *CHCHD2* mutations (Liu et al., 2020). Another study reported widespread α-syn pathology in an autopsied case harboring the *CHCHD2* T61I mutation; *CHCHD2* mutation affected α-syn stability, and loss functional CHCHD2 resulted in the accumulation of α-syn (Ikeda et al., 2019). Therefore, mitochondrial dysfunction induced by *CHCHD2* mutation promotes α-syn aggregation, leading to further mitochondrial damage, and is likely to the basis of CHCHD2 pathogenicity.

### Mitochondrial Dysfunction in Autosomal Recessive Parkinson’s Disease

#### PARKIN

Parkin is a cytoplasmic E3 ubiquitin ligase with an N-terminal ubiquitin-like domain (UBL) and a C-terminal ubiquitin ligase domain; the protein performs autoubiquitination and ubiquitination of proteins for proteasomal degradation. Parkin is encoded by *PARK2* and is composed with 465 amino acids. Under physiological conditions, parkin is localized to the cytoplasm; it is selectively recruited to the impaired mitochondria under stress conditions where it mediates phagocytosis and subsequent degradation of the mitochondria (Koyano et al., 2019). A loss-of-function mutation in *PARK2* is the most common cause of early-onset autosomal recessive PD.

Parkin is recruited when PTEN-induced putative kinase 1 (PINK1) accumulates at the depolarized OMM. Subsequently, parkin phosphorylation and activation lead to the ubiquitination of OMM proteins and the recruitment of autophagy receptors to mitochondria to initiate mitophagy, see the review by Malpartida et al. (2021). The mutations G12R, R33Q, and R42P in the UBL domain of parkin significantly reduced the ability of PINK1 to phosphorylate the protein (Aguirre et al., 2018). Furthermore, G12R and T55I mutations in the UBL domain increased parkin autoubiquitination, suggesting that these mutations accelerate parkin degradation (Aguirre et al., 2018). Increasing evidence has demonstrated that parkin is essential for the regulation of mitochondrial fission and fusion. Parkin interacts with and ubiquitinates the mitochondrial fusion proteins MFN1/2 (McLelland et al., 2018). It also ubiquitinates the mitochondrial fission protein DRP1 to stimulate its proteasome-dependent degradation instead of mitophagy (Wang et al., 2011a). In Drosophila expressing mutant parkin, the mitochondria of dopaminergic neurons were observed to be swollen (Greene et al., 2003). In addition, α-syn-mediated mitochondrial dysfunction was further aggravated in parkin knockout mice (Stichel et al., 2007).

#### PINK1

PTEN-induced putative kinase 1 (PINK1) is a serine-threonine kinase targeted to mitochondria that is associated with early-onset autosomal recessive PD. Under physiological conditions, due to the existence of the MTS sequence in PINK1, PINK1 translated in the cytoplasm is targeted to the mitochondria. The mitochondrial surface receptor translocase of outer mitochondrial membrane 20 (TOM20), with the help of TOM22 and TOM70, recognizes the MTS sequence and guides PINK1 into the translocation pore formed by TOM40 and transfers it to the Tim23 complex in the IMM (Rasool et al., 2022). Following PINK1 translocation, the IMM-localized presenilin-related diamond-like (PARL) protease cleaves PINK1 to produce PINK1 fragments approximately 52 kDa in size, then releases them into the cytoplasm (Lysyk et al., 2020). Remarkably, the transport of PINK1 into mitochondria by the TOM40 and TIM23 complex is not the only pathway of transport for this protein. Studies have shown that PINK1 can be degraded by proteasomes in the mitochondrial matrix through the mitochondrial protein transport shuttle mechanism (Thomas et al., 2014).

*PINK1* mutations are associated with loss of its kinase function. Growing evidence indicates that PINK1 acts as a crucial regulator of mitochondrial quality control through stabilization of cristae structure, phosphorylation of chaperone proteins, and regulation of mitophagy (Goncalves and Morais, 2021). The mitochondrial targeting and kinase activity of PINK1 are essential for mitochondrial localization of parkin. PINK1 accumulates on impaired mitochondria, marking them for degradation. Some PINK1 substrates have been reported to promote mitophagy by altering mitochondrial transport or dynamics. A study indicated that PINK1 phosphorylated mitochondrial Rho GTPase Miro1 at Ser156 to activate its parkin-mediated proteasomal degradation, impairing mitochondrial motility (Wang et al., 2011b). Another study reported that PINK1 phosphorylated MFN2 at Thr111 and Ser442 to induce MFN2 association with parkin and cause ubiquitination and subsequent degradation of MFN2 (Chen and Dorn, 2013). PINK1 also participated in the degradation of OMM proteins with the help of parkin, a process which is important for renewing mitochondrial surface proteins. PINK1 may phosphorylate other targets unrelated to the PINK1/parkin pathway; PINK1 can directly phosphorylate tumor necrosis factor receptor-associated protein 1 (TRAP1) in the IMS, and activated TRAP1 plays a vital role in protein folding and decreasing ROS production (Kadye et al., 2014).

#### ATP13A2

*ATP13A2* encodes the lysosomal multiple transmembrane P5B-type cation-transporting ATPase ATP13A2. Mutations in *ATP13A2* can cause early-onset autosomal recessive PD with dementia, pyramidal degeneration, and Kufor-Rakeb syndrome (KRS) (van Veen et al., 2020). Because the structure of ATP13A2 is similar to other proteins in the 5P-type ATPase family, it is also considered to be a cation pump. Several metal ions have been reported as its potential substrates. Ionic manganese (Mn^2+^) is a widely studied cation and a known environmental risk factor for PD. Studies have shown that silencing *ATP13A2* increased the toxicity of high-dose Mn^2+^ in primary neuronal cultures and mammalian cell lines (Tan et al., 2011). Furthermore, Zn^2+^ was also reported to interact with a peptide of ATP13A2. It has been reported that ATP13A2 has a hydrophobic N-terminal domain that specifically recognizes lipid signals that can enhance its protective role against mitochondrial stress (Holemans et al., 2015). Mutations in Catp-6 (an ortholog of the human *ATP13A2* gene) found in *Caenorhabditis elegans* cause defects in autophagy and lysosomal function and exhibit characteristic PD phenotypes, including dysregulation of iron metabolism (Anand et al., 2020). In addition, Catp-6/*ATP13A2* mutation impairs mitochondrial function in *C. elegans* through dysregulation of iron metabolism, and the mitochondrial dysfunction can be rescued by mitochondrial induction or iron chelation (Anand et al., 2020). ATP13A2 is involved in cellular α-syn multimerization, loss of ATP13A2 induces α-syn multimerization at the lysosomal membrane through impairing membrane integrity (Si et al., 2021).

#### PLA2G6

*PLA2G6* encodes calcium-independent phospholipase A2β protein (iPLA2β), an enzyme that selectively hydrolyzes glycerophospholipids and participates in diverse cellular functions including mitochondrial function, release of lipid mediators, cell growth, apoptosis, and calcium signaling (Ong et al., 2010; Morrison et al., 2012). Mutation of *PLA2G6* is associated with neurodegenerative disorders in humans, such as autosomal recessive neuroaxonal dystrophy and early-onset parkinsonism in humans (Lin et al., 2018). In *PLA2G6* knockout mice, ultra-structurally abnormal mitochondria with the inner membrane degeneration were reported in spinal cord neurons (Sumi-Akamaru et al., 2015), while knockout of the *iPLA2-VIA* gene in Drosophila (Drosophila homolog of *PLA2G6*) caused locomotor deficits and mitochondrial abnormalities, including defective morphology, decreased production of ATP, and mitochondrial respiratory chain dysfunction (Kinghorn et al., 2015). Loss of *PLA2G6* was confirmed to be closely associated with elevated mitochondrial lipid peroxidation (Kinghorn et al., 2015) as high levels of cardiolipin, a mitochondria-specific phospholipid, were observed in PLA2G6 knockout mice. Moreover, overexpression of iPLA2β significantly attenuated palmitate-induced mitochondrial damage through the cardiolipin/OPA1 pathway in MIN6 cells (Li et al., 2019). These studies suggest that loss of *PLA2G6* leads to membrane remodeling and mitochondrial dysfunction. Furthermore, the observation of strong α-syn expression in both *PLA2G6* knockdown neuroblastoma cells and *PLA2G6* mouse neurons indicated that *PLA2G6* deficiency resulted in the induction of endogenous α-syn in neurons (Sumi-Akamaru et al., 2016). Another study, using a Drosophila model, suggested that PD-associated iPLA2-VIA was required for the survival of DA neurons and α-syn stability through membrane remodeling (Mori et al., 2019).

#### DJ-1

*DJ-1* encodes a multifunctional protein composed of 189 amino acids, acts as an antioxidant and sensor of oxidative stress. *DJ-1* mutations lead to early-onset autosomal recessive PD. However, the specific role of DJ-1 in PD is still not clear. Early research using transmission electron microscopy found that DJ-1 was localized to mitochondria (Zhang et al., 2005). Mitochondria are the main producers for ROS. Excessive ROS induced DJ-1 to rapidly localize to mitochondria, suggesting that mitochondria may be the site of the neuroprotective activity of DJ-1; one possible mechanism is the direct interaction of DJ-1 with mitochondria to protect against ROS. Studies have shown that DJ-1 is a key regulator of mitochondrial integrity and dynamics (Liu et al., 2019; Bankapalli et al., 2020). Some studies have also described the possible role of DJ-1 in endoplasmic reticulum (ER) and mitochondrial calcium homeostasis (Liu et al., 2019). Decreased complex I activity, reduced mitochondrial membrane potential, increased ROS production, and altered mitochondrial morphology were observed in PD patients with *DJ-1* mutations and in *DJ-1* knockout mice (Xu et al., 2018; Liu et al., 2019). *DJ-1* knockout increased mitochondrial uncoupling and vulnerability in 6-hydroxydopamine (6-OHDA) PD model, while overexpression of DJ-1 reduced the MPTP-mediated loss of SNpc neurons. *DJ-1* knockdown resulted in mitochondrial fragmentation; this phenotype could be partially reversed by overexpression of PINK1 or parkin, or inhibition of DRP1 (Thomas et al., 2011). Furthermore, *DJ-1* knockout reduced the expression of mitochondrial uncoupling proteins UCP4 and UCP5, and impaired their calcium-induced uncoupling function (Surmeier, 2018). Notably, DJ-1 is also involved in inhibition of α-syn aggregation by regulating chaperone-mediated autophagy (Xu et al., 2017).

#### FBXO7

F-box domain of protein 7 (FBXO7), which contain 522 amino acids, is predominantly localized to the cytoplasm. FBXO7 has an N-terminal ubiquitin-related (UBR) domain that participates in the regulation of mitochondrial quality control through interaction with parkin (Zhou et al., 2016). Mutation in *FBXO7* causes early-onset autosomal recessive PD. FBXO7 and parkin regulate mitophagy in the same pathway, indicating that FBXO7 is necessary for successful parkin recruitment. FBXO7 promotes parkin recruitment to the impaired mitochondria, stabilizes PINK1, ubiquitinates MFN1, and facilitates mitophagy (Burchell et al., 2013; Huang et al., 2021). Wild type FBXO7, rather than mutant FBXO7, can rescue the parkin mutant-induced phenotype and mitophagy impairment in Drosophila (Burchell et al., 2013). In general, FBXO7 is involved in mitochondrial turnover, in neuroprotection, acts as a contributor to PD.

## Factors Affecting Mitochondrial Impairment in Sporadic Parkinson’s Disease

### Environmental Factors

Environmental factors were largely reported as exposures, and included post-encephalitic infection, pesticides, heavy metals, and dietary exposure (De Miranda et al., 2022). The neurotoxicity of 1-methyl-4-phenyl-1,2,3,6-tetrahydropyridine (MPTP) was discovered after observing symptoms of PD in drug-abusing patients who smoked pethidine analogs contaminated with MPTP (Langston et al., 1983); this discovery indicated that exposure to pesticides and other environmental chemicals could increase the risk of developing PD. An Agricultural Health Study found a positive correlation between the risk of PD and exposure to pesticides known to inhibit mitochondrial complex I or lead to oxidative stress (Tanner et al., 2011). MPTP and its active metabolite MPP^+^ have been widely used in experimental animals and cell models of PD, respectively. Epidemiological studies have shown that the risk of PD increased in individuals who used rotenone in agriculture or who lived in close proximity to areas where rotenone was used (Tanner et al., 2011). Rotenone exposure was shown to induce degeneration of dopaminergic neurons, neuroinflammation, α-syn accumulation, and other pathological and behavioral alterations in rodent models (Xue and Bian, 2015). Furthermore, rotenone can directly impair mitochondrial function by inhibiting complex I and altering mitochondrial transport. The role of environmental factors of in the pathophysiology of PD remains elusive. However, it is well known that environmental factors related to cellular phenotype can be regarded as cellular hallmarks of PD.

### Iron Accumulation and Oxidative Stress

In addition to genetic and environmental factors, there is a significant association between PD and disorders of iron metabolism. Increased brain iron of PD patients suggests that the interaction between iron and melanin may promote oxidative neuronal damage (Ndayisaba et al., 2019). Mitochondria act as the major iron recipients (Cerri et al., 2019). Iron ions in the SN may catalyze the conversion of H_2_O_2_ to highly active hydroxyl radicals, generated during the breakdown of DA, which may lead to oxidative damage (Jiang et al., 2017). Ferroptosis is a regulated, iron-dependent cell death pathway that involves a decrease in glutathione peroxidase 4 (GPX4) activity and the accumulation of lipid peroxide (Mahoney-Sanchez et al., 2021). Ferroptosis has several characteristics similar to PD pathophysiology and has been confirmed to be involved in the pathogenesis of PD. Mice exposed to iron produced parkinsonian phenotypes with loss of neurons in the SN and depletion of DA in the striatum (Kaur et al., 2007). It is known that MPTP, rotenone, and paraquat can inhibit the activity of mitochondrial complex I, which is associated with iron deposition (Munoz et al., 2016). Furthermore, an iron chelator can reduce the toxicity of these neurotoxin. Studies have shown that iron-mediated oxidative stress can promote α-syn aggregation (Angelova et al., 2020), indicating a close relationship between oxidative stress, α-syn, iron, and PD.

### Mitochondrial DNA and Polymorphisms in Parkinson’s Disease

The mitochondrion is the only organelle with its own genome (mtDNA) in the cell. mtDNA is composed of multiple copies of circular, double-stranded molecules; it encodes 13 essential polypeptides in the respiratory chain, 2 ribosomal RNAs, and 22 transfer RNAs that are essential for mitochondrial protein synthesis. mtDNA is continuously damaged, which can eventually lead to mutations and mitochondrial dysfunction. Although mtDNA deletions were detected in elderly control subjects, there was an age-related accumulation of deletions that was accelerated in the striatum of PD patients (Ikebe et al., 1990; Manfredi, 2006); this association indicated that the mtDNA deletions contributed to the pathological process of PD. Increasing research has observed accumulation of deletions, mutations, and rearrangements of mtDNA in animal models of PD and in PD patients. A high level of deleted mtDNA found in the SN dopaminergic neurons of idiopathic PD patients was related to respiratory chain dysfunction (Grunewald et al., 2016).

Specific mitochondrial haplogroups are associated with either a lower or higher risk of developing PD. A systematic investigation in 2003 found that haplogroups J and K were associated with significantly reduced risk of developing PD and that this protective effect may have been associated with the single-nucleotide polymorphism 10398G, a common variant in the ND3 subunit of complex I (van der Walt et al., 2003). A two-stage association study confirmed that haplogroups J, K, and T were associated with a reduction in risk of developing PD; however, the super-haplogroup HV was associated with an increased risk of PD. mtDNA polymerase gamma (POLG), encoded by nuclear DNA (nDNA), was shown to participate in the replication, synthesis, and repair of mtDNA (Filosto et al., 2003). Mutations in the POLG gene caused multiple mtDNA deletions and PD phenotypes. In summary, it can be speculated that mtDNA haplogroups may have an antagonistic effect and influence susceptibility to age-dependent neurodegenerative diseases.

### Mitochondria and Epigenetic Modifications in Parkinson’s Disease

Multiple lines of evidence suggest that pathological processes in PD may result from a combination of both genetic and environmental factors (epigenetic modifications). Such modifications may damage mtDNA and impair mitochondrial function, while older individuals who experience prolonged stress are more likely to experience multiple molecular changes, such as the release of endogenous neurotoxins, higher oxidative stress levels, and epigenetic modifications. Epigenetic modifications have been reported to induce a series of changes in gene expression and protein levels that result in mitochondrial abnormalities and neuronal apoptosis and lead to PD (Ndayisaba et al., 2019). Environmental stresses have been shown to trigger epigenetic changes that induce molecular variation and alter the normal function of loci or chromosomes (Billingsley et al., 2019). Growing numbers of studies support the role of epigenetic modifications in explaining expression differences at the mitochondrial level in PD. In addition, non-coding RNAs (ncRNAs) can regulate many genes at the transcriptional level, despite not being translated into proteins. Their functions have been detected in the mitochondria, while ncRNAs such as microRNAs (miRNAs) (Zhang et al., 2021), long non-coding RNAs (Kopp et al., 2019), and some circular RNAs (Shao and Chen, 2016) have been shown to play multiple roles in many of the underlying mechanisms of PD pathogenesis. Observations of various epigenetic changes in mtDNA have received considerable attention. Epigenetic modifications of mtDNA, such as methylation and hydroxymethylation, are related to various environmental factors (including oxidative stress, therapeutic strategies, and aging) that induce parkinsonism (Byun and Baccarelli, 2014). One study reported increased mtDNA serum concentrations in female PD patients and demonstrated detectable cytosine-phosphate-guanine (CpG) methylation sites in the mitochondrial epigenome in platelet-derived mtDNA (Sharma et al., 2021). Furthermore, histone modifications in mitochondrial impairment are associated with PD pathology. Genome-wide histone acetylation analysis in PD patients revealed increased acetylation at multiple histone sites (H2B, H3, and H4), with the most notable change observed for H3K27, strongly suggesting that abnormal histone modification and altered transcriptional regulation are involved in the pathophysiology of PD (Toker et al., 2021). Notably, epigenetic modifications can also be affected by dietary nutrients, resulting in dynamic changes over the lifespan of an organism (Milosevic et al., 2021). Overall, mitochondria are currently attracting an increasing amount of attention in the field of PD epigenetics, providing a potential strategy for PD.

## Mitochondrial Quality Control in Parkinson’s Disease

**FIGURE 1:**
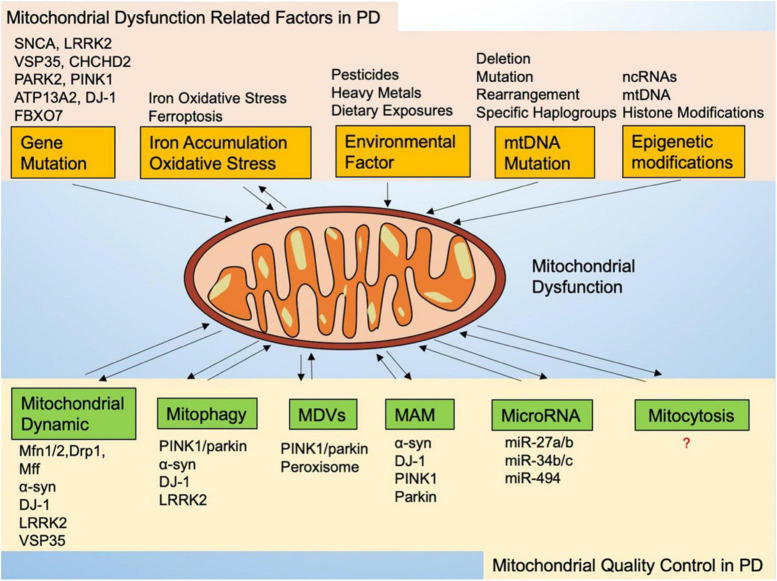
Mitochondrial dysfunction-related factors and mitochondrial quality control in Parkinson’s disease.

Quality control mechanisms are essential to maintaining a healthy mitochondrial network and cell homeostasis, especially to dopaminergic neurons. Mitochondria exhibit broad quality control mechanisms, regulated primarily by mitochondrial dynamics and mitophagy. Further elucidation of the mitochondrial quality control pathways used to protect cells may provide potential opportunities for PD treatment in cases of mitochondrial dysfunction (Figure 1).

### Mitochondrial Dynamics

Mitochondria are highly dynamic and mobile organelles, constantly undergoing membrane remodeling through fission, fusion, and turnover to maintain mitochondrial homeostasis and function. Due to neuronal polarity, there is continuous transportation and exchange of material between the cell body and the synapses. Mitochondria are also transported across the cell body and synapses to ensure that energy requirements are met between different subcellular compartments. Excessive mitochondrial fission is widely thought to be the cause of mitophagy, leading to dopaminergic neurodegeneration in PD models (Chen et al., 2021). It is believed that dysregulation of mitochondrial dynamics is linked with neuroinflammation through the induction of interleukin-10 (IL-10) and reduction of tumor necrosis factor-α (TNF-α) secretion (de Oliveira et al., 2021). In response to cellular stress, a pathway for mitochondrial antigen presentation (MitAP) triggers an autoimmune response through extraction of self-antigens from mitochondria *via* MDVs and presentation at the cell surface (Roberts and Fon, 2016); PINK1 and parkin, actively repress MitAP by inhibiting this pathway in dysfunctional mitochondria (Matheoud et al., 2016). In fact, persistent activation of microglia in the brains of PD patients has been demonstrated (Gerhard et al., 2006). In PD, parkin deficiency results in the accumulation of impaired mitochondria and increased MitAP, which triggers and exacerbates central and peripheral inflammation. The pathway for MitAP relies on PINK1 and parkin, providing a bridge between mitochondrial dynamics and neuroinflammation in PD.

Mitochondrial fusion and fission are regulated by several proteins. Mitochondrial fusion is mainly mediated by MFN1, MFN2, and OPA1 (Rodrigues and Ferraz, 2020). Mitochondrial fission is regulated mainly by cytoplasmic DRP1, fission1 (FIS1), and mitochondrial fission factor (MFF) (Zhou et al., 2020). Increased fusion or fission leads to mitochondrial elongation or fragmentation, resulting in impaired mitochondrial function. It is difficult to study mitochondrial morphology in PD patients because the ultrastructure of post-mortem brain tissue of PD patients are not well preserved; however, PD-related neurotoxins can cause DRP1-dependent mitochondrial fragmentation *in vivo* and *in vitro*, indicating that mitochondrial fusion and fission are involved in PD (Santos and Cardoso, 2012). Studies have shown that DJ-1 deficiency can cause mitochondrial fragmentation that can be rescued by PINK1 and parkin (Thomas et al., 2011). Notably, other research has indicated that PINK1 and parkin participate in the control of mitochondrial dynamics (Scarffe et al., 2014). α-Syn is localized to the mitochondria, has a direct effect on the regulation of mitochondrial morphology (Vicario et al., 2018), and affects mitochondrial axonal transport and synaptic function (Koch et al., 2015). Furthermore, LRRK2 and VSP35 are thought to regulate mitochondrial morphology and transport (Mir et al., 2018), suggesting that mutant genotypes associated with familial PD may represent the upstream mechanism regulating mitochondrial morphology and transport.

### Mitophagy

There are three main forms of autophagy: microautophagy, molecular chaperone mediated autophagy (CMA), and macroautophagy. Mitophagy is a form of macroautophagy, a selective degradation of impaired mitochondria that limits the accumulation of dysfunctional mitochondria in the cell. It is an essential process in maintenance of the health of mitochondria. Increasing evidence has shown that neurodegeneration is accompanied by abnormal mitophagy.

In 2008, researchers discovered the relationship between the PD-related protein parkin and mitophagy (Narendra et al., 2008); it is considered a milestone study of mitophagy. Several subsequent studies have shown that PINK1, another protein associated with PD, is also involved in mitophagy (Clark et al., 2021; Goncalves and Morais, 2021). The PINK1/parkin pathway is the most widely studied mechanism in mitophagy. Under normal conditions, PINK1 is rapidly degraded after cleavage by PARL in low levels on the surface of mitochondria. Dissipation of mitochondrial membrane potential caused by mitochondrial depolarization, protein misfolding, or ROS can impair this degradation process; dissipation is considered the main event that initiates of mitophagy, causing the accumulation of PINK1 on the surface of mitochondria and the recruitment of parkin (Wang et al., 2021b). PINK1 phosphorylates MFN2 and ubiquitin, which in turn causes recruitment of parkin (McLelland et al., 2018). Several proteins ubiquitinated by parkin on the OMM then form phosphoubiquitin chains, which recruit autophagy receptors and induce mitochondrial autophagy. Loss of PINK1 or parkin function causes significant mitochondrial disease and degeneration of dopaminergic neurons (Morais et al., 2014; Cartelli et al., 2018). Mitophagy mediated by PINK1 and parkin also involves changes in mitochondrial dynamics. Both MFN1 and MFN2 are targets of parkin and are marked for degradation (Gegg et al., 2013), which can inhibit mitochondrial fusion. Furthermore, mitochondrial fission facilitates mitophagy by producing smaller organelles that are more easily engulfed by autophagosomes. Excessive fission caused by loss of parkin or PINK1 can be countered by MFN2 and OPA1 or by dominant negative mutations of the fission protein DRP1 (Giannoccaro et al., 2017). Interestingly, the PINK1/parkin pathway can prevent mitochondrial movement and isolate damaged mitochondria before clearance. PINK1 was reported to phosphorylate mitochondrial motility related protein Miro and activate proteasomal degradation of Miro in a parkin-dependent manner (Shlevkov et al., 2016). Mitochondrial dysfunction and impairment of autophagy are key factors in the pathogenesis of PD. In addition to the PINK1/parkin pathway, LRRK2, α-syn, DJ-1, and other PD-related proteins have been reported to be involved in mitochondrial quality control or mitophagy, see Verstraeten et al.’s (2015) review. G2019S substitution of LRRK2 mutation weakened the interaction between parkin and DRP1, leading to interference with PINK1/parkin mitophagy (Bonello et al., 2019). DJ-1 promoted mitophagy to respond to oxidative stress, acting simultaneously with the PINK1/parkin pathway (Thomas et al., 2011). Mitophagy plays a crucial role in the pathogenesis of PD. Thus, a thorough study of how these genes are involved in the regulation of mitophagy is essential for understanding the pathogenesis of PD and for the development of new therapeutic strategies.

### Mitochondrial-Derived Vesicles

Mitochondrial-derived vesicles, first reported in 2008, is involved in a repair mechanism that transports damaged mitochondria or oxidized proteins (Neuspiel et al., 2008). MDVs are composed of a double membrane with a diameter of 70–150 nm; they bud off from the mitochondria. In contrast with mitophagy, the MDVs pathway handles mildly damaged mitochondria and is independent from mitochondrial depolarization, fission, and autophagy.

Two different types of MDVs have been studied extensively. The first type of MDVs is PINK1/parkin-dependent and targets cargo proteins directly to lysosomes (McLelland et al., 2016). Notably, this process acts independently from autophagy and the oxidized cargo protein is degraded directly in the lysosome (Soubannier et al., 2012). These MDVs act as a defense mechanism to reduce mild mitochondrial damage below the mitophagy threshold. To rapidly respond to ROS production and oxidative stress, a local PINK1/parkin pathway is activated for exporting damaged proteins to the lysosome; disruption of this pathway may influence the ability of mitochondria to remove oxidized proteins, potentially exacerbating mitochondrial dysfunction in PD (McLelland et al., 2014). The second type of MDVs is targeted from mitochondria to peroxisomes (Soubannier et al., 2012). The only protein known to be able to transport vesicles to peroxisomes is membrane anchored protein ligase (MAPL) reviewed in Sugiura et al. (2014). Research has shown that this process is associated with a reverse complex involved in the transport of cargo proteins from the endosome to the Golgi apparatus (Braschi et al., 2010). Interestingly, another PD-linked gene, VPS35, has been found in the reverse transcription complex with MAPL; it regulates the formation of MDVs and participates in directing MDVs to peroxisomes. MDVs mediate VPS35-dependent mitochondrial DRP1 turnover through VPS35-DRP1 interaction and PD-linked mutations in VPS35 cause the accumulation of DRP1, leading to fragmentation of the mitochondrial network (Wang et al., 2016). In general, MDVs-mediated mitochondrial quality control mechanisms ensure organelle homeostasis before triggering more mitochondrial degradations.

### Mitochondria-Associated Membranes

Mitochondria-associated membranes are a specialized domain that interface with 5–20% of the OMM and ER membranes (Wang et al., 2021a). The physical tethering of ER to mitochondria was confirmed through transmission electron microscopy in the early 1960s. The ER and mitochondria were connected by tethers at a physical distance of 10–25 nm (Csordas et al., 2006; Liu et al., 2022). Due to variation in the quantity and molecular composition of MAMs, they are considered dynamic structures. Increasing evidence has indicated that MAMs regulated several cellular processes, including Ca^2+^ homeostasis, phospholipid exchange, mitochondrial biogenesis, autophagy, and mitochondrial dynamics (Wu et al., 2016; Dia et al., 2020; Yeo et al., 2021).

Several PD-related proteins, such as α-syn, parkin, PINK1, DJ-1, VPS35, and LRRK2, have been confirmed to alter the ER-mitochondrial signaling and influence Ca^2+^ balance. Furthermore, PD-linked mutations have been shown to impair MAMs. Abnormally accumulated α-syn can alter the number of the ER-mitochondrial contact sites and alter the transport of Ca^2+^ (Paillusson et al., 2017); α-syn interaction with vesicle-associated membrane protein-associated protein B (VAPB) in the MAMs protein complex interferes with the contact between VAPB and tyrosine phosphatase interacting protein 51 (PTPIP51) of MAMs. α-Syn is localized to the MAMs. The aggregation of α-syn weakened the interaction between α-syn and MAMs, altering mitochondrial morphology and mitochondrial intracellular distribution, see the exciting review by Rowland and Voeltz (2012). α-Syn mutations can also impair tethering and weaken the association between mitochondria and the ER (Cali et al., 2019). It has further been suggested that aggregated α-syn represents the initiating factor that disturbs mitochondrial dynamics during the pathogenesis of PD. In addition, parkin knockdown has been shown to result in fragmented mitochondria and reduce the number of the ER-mitochondria contact sites in the SH-SY5Y cell model (Cali et al., 2013). DJ-1 overexpression increased the ER-mitochondria connection and DJ-1 knockout resulted in significant functional impairment of MAMs both *in vitro* and *in vivo* (Liu et al., 2019). Alterations of MAMs in different PD models are summarized exhaustively in Gomez-Suaga et al.’s (2018) review. Although MAMs function is impaired in PD, it is still unclear whether MAM dysfunction is the cause or effect of the pathogenesis of PD; however, it is clear that the dysregulation of MAM can facilitate neuronal death.

### MicroRNA and Mitochondrial Anomalies in Parkinson’s Disease

MicroRNAs (miRNAs) are ncRNAs, 18–25 bases in length, that serve as regulators that affect stability and translation of mRNA targets by directing RNA-induced silencing complex (RISC) to the 3′-UTR, more details see Bartel’s (2018) review. Increasing evidence has revealed the association between miRNA and the pathogenesis of PD (Rostamian Delavar et al., 2018). It has been reported that levels of several miRNAs are altered both in PD models and in the brain tissue of PD patients (Viswambharan et al., 2017; Rostamian Delavar et al., 2018). Most of the altered miRNAs in PD patients are directly associated with mitochondrial function reviewed in Sun et al. (2019).

It has been reported that miR-133b is downregulated in the midbrain of PD patients, which indirectly decreases the expression of vesicular monoamine transporter 2 (VMAT2) by downregulating paired-like homeodomain transcription factor 3 (Pitx3) and altering the expression of dopamine transporter (DAT) through the same process that alters VMAT2 expression (Hwang et al., 2009). However, MiR-137 and miR-491 have been shown to reduce DAT expression, thereby influencing neuronal DA transport *in vitro* (Jia et al., 2016). The altered expression of these miRNAs is also associated with oxidative stress in PD. MiR-27a and miR-27b were found to regulate mitochondrial quality control by inhibiting the expression of PINK1 (Kim et al., 2016). MiR-34b and miR-34c are downregulated in the SNpc of PD patients. MiR-34 was found to reduce neuronal viability through mitochondrial dysfunction and the production of ROS *in vitro* (Minones-Moyano et al., 2011; Polytarchou et al., 2020). Further studies have suggested that the reduction in expression of miR-34b and miR-34c is related to the expression of DJ-1 and parkin, although the underlying mechanism is still unclear (Polytarchou et al., 2020). According to reports, several miRNAs regulate the expression and accumulation of α-syn, such as miR-7, miR-34b/c, and miR-19a-3p (Consales et al., 2018; Zhou et al., 2019; Zhang et al., 2021); their alterations may promote α-syn-mediated neurotoxicity. Abnormal expression of miRNA can be triggered by long-term environmental stressors, such as oxidative stress, toxins, or pathogens that induce both physiological and pathological stress. The various environmental stresses that humans experience throughout their lives may alter transcriptional regulation, consequently inducing mitochondrial dysfunction and eventually lead to sporadic PD.

### Migrasome-Mediated Mitochondrial Quality Control

In 2015, researchers discovered a pomegranate-like structure with a diameter of about 2 μm outside of cells, named the “migrasome,” which formed on the retraction fibers of migrating cells (Ma et al., 2015). Mass spectrometry revealed that tetraspanins were enriched in migrasomes, and studies have confirmed the necessity of tetraspanins and cholesterol for migrasome formation through live-cell experiments and reconstituted membrane systems (Huang et al., 2019). When migrating cells were under mild mitochondrial stress, they disposed of impaired mitochondria through mitocytosis, which is a migrasome-mediated mitochondrial quality control process (Jiao et al., 2021). Under normal conditions, inhibiting mitocytosis resulted in decreased mitochondrial membrane potential and respiratory function; enhancing mitocytosis had a protective effect, preventing the loss of mitochondrial membrane potential and the impairment of mitochondrial respiration induced by mitochondrial stressors (Jiao et al., 2021). *In vivo* studies confirmed that mitocytosis was beneficial for maintaining a healthy mitochondrial network in highly migratory neutrophils. Although migrasomes have been detected in the infarcted brain parenchyma of stroke patients and described as a new mechanism in the pathophysiology of acute stroke, further research regarding these structures is needed (Schmidt-Pogoda et al., 2018). In the aged brain, subventricular zone neurogenesis is reduced due to a decrease in the number of neural stem cells (Capilla-Gonzalez et al., 2014). Because the glial tube is maintained by neuroblast secretion of slit guidance ligand 1 (Slit1), a small number of neuroblasts may not produce enough Slit1 to maintain the most effective migration route (Kaneko et al., 2010). It is unclear whether mitocytosis is involved in the pathogenesis and progression of PD, and further research into mature dopaminergic neuron migration and mitocytosis would be essential to the study of PD pathogenesis.

## Mitochondrial Therapeutics for Parkinson’s Disease

The mechanisms of PD pathology related to mitochondrial dysfunction have motivated the development of novel anti-PD therapies. Translating promising mitochondrial targets to clinical use in familial PD patients and further to sporadic PD patients is a daunting challenge for PD researchers. To this end, various strategies have been devised to improve mitochondrial function in PD.

### Gene Therapy

In 1972, gene therapy was initially described as a method of “using good DNA to replace bad DNA,” see Friedmann and Roblin’s (1972) review. With the rapid developments in science and technology, the basic principles of gene therapy persist, but the process has become more complex. The most common strategy is to use viral vectors, such as lentivirus and recombinant adeno-associated virus (AAV), to effectively target delivery of genetic material into cells to regulate the expression of specific genes. Several gene therapy targets have been identified in PD: restoration of DA synthesis, enhancement of neurotrophic support, and modulation of the interaction between different functional brain nodes. Gene therapy in PD is designed to target key elements in the mitochondrial pathway that prevent neurodegeneration. PGC-1α is a transcriptional coactivator that positively regulates expression of several genes required for mitochondrial biogenesis and antioxidant response; the level of PGC-1α is decreased in PD patients (Jo et al., 2021); therefore, PGC-1α has become a promising therapeutic target for neuroprotection in PD interventions. In addition, CRISPR-CAS9 gene editing technology can correct gene mutations that result in focal disease activity and may also alter the DNA of germ cells to protect offspring from familial PD, for more information refer to the two reviews by Yang et al. (2016) and Karimian et al. (2020).

### Antioxidant Therapies

During the progression of PD, neurons were shown to exhibit mitochondrial dysfunction with decreased production of ATP and reduced expression of the respiratory chain complex, coinciding with increased ROS generation. It is well known that damaged mitochondria produce greater amounts of ROS than healthy mitochondria. Furthermore, neuronal mitochondria are more vulnerable to oxidative stress and have a slow turnover (Yang et al., 2020). Free radicals produced by oxidative stress affect the structure and function of neural cells, which may disrupt antioxidant mechanisms and ultimately lead to neurodegeneration.

Resveratrol was reported to have antioxidant effects and improved movement disorders in a MPTP mouse model (Guo et al., 2016). Resveratrol has been shown *in vitro* to act through the AKT/GSK-3β pathway against MPP^+^ induced mitochondrial dysfunction and apoptosis (Xiong et al., 2020). Although coenzyme Q10 (CoQ10) is an antioxidant that has been shown to enhance the activity of complex I and II in the electron transport chain, a meta-analysis reported that CoQ10 supplementation did not slow down the functional decline of PD patients, nor did it provide any symptomatic improvement (Negida et al., 2016). Researchers have also proposed that only a certain subset of PD (“mitochondrial form of PD”) can benefit from CoQ10 treatment; it was reported that CoQ10 counteracted the neurotoxicity induced by MPTP and prevented the electron transfer from complex I to others (Cleren et al., 2008). Furthermore, compared with other untargeted antioxidants, MitoQ was more effective in preventing mitochondrial oxidative damage (Feniouk and Skulachev, 2017). It was demonstrated that MitoQ had a protective effect on 6-OHDA-induced mitochondrial dysfunction in PD models *in vivo* and *in vitro*; the possible mechanism involved PGC-1α-mediated increase of MFN2 expression (Xi et al., 2018). Saffron is considered to have multiple health-promoting effects. Saffron extracts and its bioactive components have antioxidant properties, which can restore the activity of antioxidant enzymes such as superoxide dismutase (SOD), glutathione S-transferase (GST), and catalase, increasing the level of glutathione and total thiol, reducing ROS, and providing a potential therapy for improving the motor symptoms of PD (Rahaiee et al., 2015; Inoue et al., 2021). N-acetylcysteine (NAC) is a powerful scavenger of free oxygen radicals, which keeps sulfhydryl groups in a reduced state and restores the level of neuronal glutathione (GSH) (Shahripour et al., 2014). Because the death of dopaminergic neurons is related to oxidative stress and GSH depletion, and it has been reported that normalization of GSH levels can reduce oxidative stress and cell death; therefore, regulating neuronal GSH metabolism has potential therapeutic significance for PD. Interestingly, some antioxidants, such as resveratrol and vitamin E, are able to regulate DNA methylation and histone acetylation by inhibiting DNA methyltransferase and histone deacetylase activity (Coupland et al., 2014; Li et al., 2021). Oxidative stress and mitochondrial dysfunction are closely associated with the pathophysiology of PD. Prevention of the formation of excessive ROS and antioxidant therapy to ameliorate mitochondrial function in a timely and effective manner represent promising strategies for treating PD.

### Improving Mitochondrial Biogenesis

Mitochondrial biogenesis involves complex processes and requires the joint participation of the mitochondrial genome and the nuclear genome to maintain mitochondrial homeostasis. The biogenesis of a large number of functional mitochondria may reduce the production of ROS. A genome-wide study showed that the expression of PGC-1α, the main controller of mitochondrial biogenesis, was reduced in PD models compared with controls, which confirmed the role of mitochondrial biogenesis in the pathogenesis of PD (Imam et al., 2013; Yazar et al., 2021). A recent study showed that parkin-knockout DA neurons had mitophagy defects that mainly resulted from mitochondrial biogenesis defects driven by parkin interacting substrate (PARIS) upregulation and subsequent PGC-1α downregulation (Pirooznia et al., 2020). In addition to regulating mitophagy, parkin is also involved in controlling mitochondrial biogenesis (Kumar et al., 2020). Therefore, inducing or improving mitochondrial biogenesis should be considered a therapeutic target of neuroprotective methods for the treatment of PD. Although other approaches can be targeted to regulate mitochondrial biogenesis, targeting of the AMPK-SIRT1-PGC-1α axis would be most important pathway to enhance mitochondrial biogenesis (Chandra et al., 2019). It has been demonstrated that the activation of PGC-1α had neuroprotective effects in different mouse models of PD. Bezafibrate, acting as pan-PPAR agonist, upregulated PGC-1α levels and exhibited protective effects in mouse models of neurodegenerative disease (Johri et al., 2012). In addition, recent research has confirmed that a dopamine D1 receptor agonist can ameliorate mitochondrial biogenesis in a rat model of PD, and D1 agonist-mediated effects were eliminated following D1 receptor antagonist treatment (Mishra et al., 2020). It is worth mentioning that mitochondrial transplantation by supplementing exogenous mitochondria is a promising strategy in PD, refer to this interesting review by Shanmughapriya et al. (2020).

### Enhancing Mitophagy

Mitophagy is important to the pathophysiology of PD. An ultrastructural study showed mitophagy accumulation in neurons of Lewy body dementia (LBD) and PD patients (Hou et al., 2018). Parkin and PINK1 genes are the pivotal causes of autosomal recessive PD and intermediate mitophagy (Gan et al., 2022). Studies have shown that celastrol reduced dopaminergic neuron death in a PD cell model and inhibited neurodegeneration in a PD animal model through upregulation of PINK1 and enhanced mitophagy (Lin et al., 2020). A drug screening experiment used a high-throughput phenotyping system on dopaminergic neurons derived from iPSCs collected from PD patients with parkin or PINK1 mutations to identify four candidates that could effectively improve the clearance of damaged mitochondria through screening 320 compounds (Yamaguchi et al., 2020). Some of the candidates were validated as they were found to ameliorate motor deficits caused by PINK1 deficiency in Drosophila and to be effective in iPSCs derived from idiopathic PD patients with impaired mitochondrial clearance. Melatonin is present in high concentrations in the mitochondria, which can prevent the oxidation of cardiolipin and help to restore mitophagy in PD (Chu et al., 2013). In addition, activators of parkin and PINK1 and inhibitors of USP30 and USP14 present promising therapeutic strategies for enhancing mitophagy (Xiang et al., 2020; Luo et al., 2021). Enhancing mitophagy is a meaningful therapeutic approach in PD (Aman et al., 2020).

### Challenges

Mitochondrial therapeutics undoubtedly have a positive impact on PD progression. However, these approaches allow only for symptomatic treatment and do not prevent the severe disability and significant reduction in quality of life that occur at later stages of disease. Various therapeutic approaches focus on the possible mitochondrial etiology of PD, as changes in the pathophysiological network of mitochondria can lead to the disease; nonetheless, the exact mechanism of the interactions between these networks is not fully understood, see Borsche et al.’s (2021) review. Clinical trials for PD have had limited success to date, and there are still no effective neuroprotective therapies to suppress or slow down the progression of PD. Lack of success in clinical trials can be attributed to an incomplete understanding of the molecular pathways and targets in PD, the heterogeneity of patient populations, a lack of adequate biomarkers to monitor drug efficacy, distinguish patient subtypes, and limited understanding of the sequence in which different cellular mechanisms lead to neuronal loss, see Lang and Espay’s (2018) review. Furthermore, PD presents many challenges to therapeutic approaches, including the difficulty of introducing therapeutics into the brain, target specificity, potential inflammatory responses, safety concerns, tolerability for geriatric patients, ethical implications, and the mechanistic heterogeneity of the disease, refer to these reviews by Jarrin et al. (2021) and Vijiaratnam et al. (2021). A major challenge for any preventative therapy is that much of the mitochondrial dysfunction in the brain of PD patients may have already occurred before the point of diagnosis during the prodromal period, see the excellent review by Schapira and Tolosa (2010). A study published in Nature in 2021 was the first to simulate a model of prophase PD, as well as the first to show that simple damage to mitochondrial function could fully simulate the pathogenesis of PD in animals. This suggested that mitochondrial dysfunction occurs before the onset of PD and that targeting mitochondria may therefore potentially provide the opportunity to slow the progress of PD at the earliest possible stage (Gonzalez-Rodriguez et al., 2021). Therefore, one of the most critical challenges is the identification of biomarkers for the prodromal stages of PD, which would allow the earlier initiation of new therapeutic strategies.

## Concluding Remarks

Currently, only symptomatic treatment of PD is available; finding strategies that can directly target the underlying mechanisms of PD or delay the progression of the disease is essential. Mitochondrial complex I dysfunction, mtDNA damage, and mitophagy dysfunction were observed in the neurons of sporadic and monogenic PD patients and in gene mutation models, which suggests that mitochondria play an important role in the pathophysiology of PD. The discovery of new mitochondrial genes associated with PD has contributed to understanding the influence of mitochondrial dysfunction on neurodegeneration and the underlying molecular mechanisms. Research on the mechanism of mitochondrial dysfunction in PD has shown promising results and has provided hope for finding an effective PD treatment in the future. However, research still needed to determine the pathogenic impact of mitochondrial dysfunction in the development of PD and the underlying mechanisms involved.

## Author Contributions

X-YG and X-HS conceived and coordinated the review. TY and YG provided the intellectual inputs. All authors read and approved the final manuscript.

## Conflict of Interest

The authors declare that the research was conducted in the absence of any commercial or financial relationships that could be construed as a potential conflict of interest.

## Publisher’s Note

All claims expressed in this article are solely those of the authors and do not necessarily represent those of their affiliated organizations, or those of the publisher, the editors and the reviewers. Any product that may be evaluated in this article, or claim that may be made by its manufacturer, is not guaranteed or endorsed by the publisher.
